# RNA trans-splicing treatment for Duchenne muscular dystrophy

**DOI:** 10.64898/2026.09.13.751281

**Published:** 2026-09-16

**Authors:** Ryan H. Hsu, Claire E. Williams, Geraldine Maier, Miriam Gullo, Karen Lettieri, Cindy J. Alvarez, Kip J. Hermann, Stella Kramer, Satchidananda Panda, Lukas C. Bachmann, Samuel L. Pfaff

## Abstract

Duchenne muscular dystrophy (DMD) is a fatal disorder caused by *dystrophin* mutations, leading to progressive muscle degeneration and cardiac failure. Although AAV-based DMD therapies with innovative designs to overcome the cargo limits of the virus are effective in animal models, these current systems may face clinical translation challenges related to efficiency, off-target effects, and immunogenicity. We developed a multi-vector RNA End Joining (REJ) system to split large genes into multiple co-delivered AAVs that engage the cell’s intrinsic spliceosome to precisely reassemble RNA segments that encode very large scar-free proteins. We show the system is efficient and has negligible off-target interactions. *In vivo* , REJ vectors expressing the adenine base editor Abe8e, mini-dystrophin Dp253, or native full-length dystrophin Dp427 each prevented muscle degeneration. Machine-learning histopathology of more than 120,000 myofibers revealed robust reductions in nuclear infiltration, improved centronucleation, and normalized hypertrophy, supported by functional, transcriptomic, and behavioral analyses. These findings establish REJ as a clinically viable AAV-based strategy for DMD that enables expression of large therapeutic proteins while avoiding potentially antigenic bacterial protein- or DNA-recombinases.

## Full Text Availability

The license terms selected by the author(s) for this preprint version do not permit archiving in PMC. The full text is available from the preprint server.

